# Male with chest pain after blunt trauma

**DOI:** 10.1016/j.acepjo.2026.100362

**Published:** 2026-04-04

**Authors:** Kahra Nix, Nicholas DiMeo, Garrett Stults, Luther Daniel, Jeffery Baker

**Affiliations:** Department of Emergency Medicine, University of Louisville, School of Medicine, Louisville, Kentucky, USA

## 1 Patient Presentation

A 63-year-old man with history of a mechanical aortic valve on warfarin presented after a motor vehicle accident. The paramedic found him still restrained but confused and complaining of chest pain. An on-scene electrocardiogram showed ST segment elevation in V1, V2, and V3. In the emergency department, his heart rate was 106 cpm, and his blood pressure was 90/50 mm Hg. Examination revealed an abrasion and tenderness over his sternum. Focused assessment of sonography in trauma (FAST) did not find free fluid. Repeat electrocardiogram showed sinus tachycardia and anterior and inferior q waves. The high-sensitivity troponin was 14,646 (ng/L).

## 2 Diagnosis: Ventricular Septal Defect

**Figure 1:**
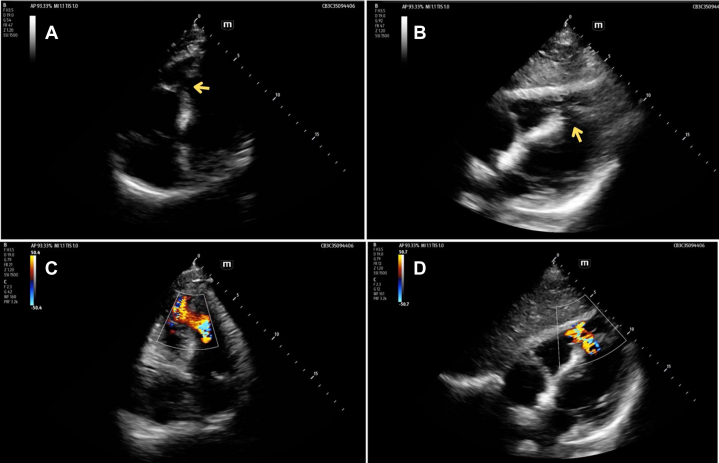
Point-of-care focused cardiac ultrasound was performed on a patient with elevated, high-sensitivity troponin after blunt chest wall trauma. The apical 4 (A) and subxiphoid (B) views show an apical ventricular septal defect (yellow arrow), apical hypokinesis, preserved ejection fraction, and equality of ventricles. Color Doppler shows turbulent flow through the defect (C and D).

**Figure 2:**
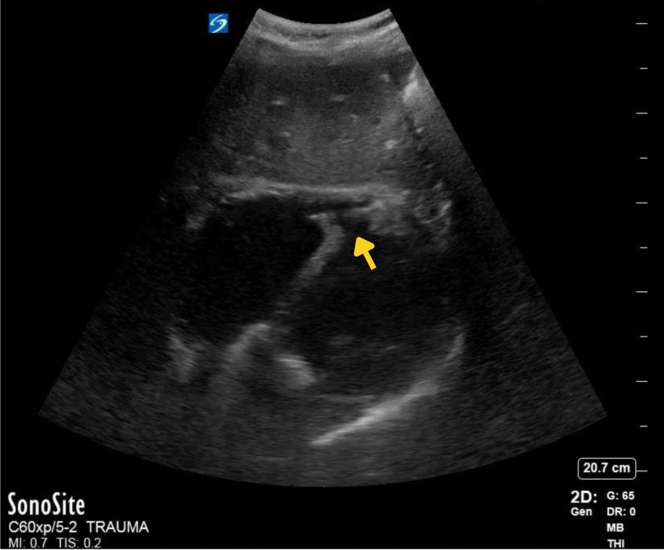
Focused assessment of sonography in trauma (FAST) was performed on a patient who presented after blunt chest wall trauma and did not show any intraperitoneal or pericardial free fluid. However, the subxiphoid FAST view showed a ventricular septal defect (yellow arrow).

Focused cardiac ultrasound, performed after the troponin resulted, revealed a ventricular septal defect (VSD) (Fig.1, Videos 1 and 2). Review of the initial FAST also showed the defect (Fig. 2). Whole-body computed tomography (CT) revealed a left first and right third and fourth rib fractures but not the VSD. A one-hour repeat troponin was 24,341 (ng/L). Transthoracic echo by cardiology confirmed the VSD. Cardiac catheterization did not show obstructive coronary artery disease or other traumatic injury. Ventricular septal rupture from blunt trauma is thought to result from compression of the heart between the sternum and spine during diastole or from a healed, congenital VSD.^1^ His normal right ventricular (RV) structure and function made it unlikely that this large VSD was pre-existing or from a previously repaired defect.^2^^,^^3^ Despite risks of RV failure and pulmonary hypertension, he elected medical management due to the mortality risk of surgery. Days later, cardiac magnetic resonance found worsening RV function.

**Video 1:**
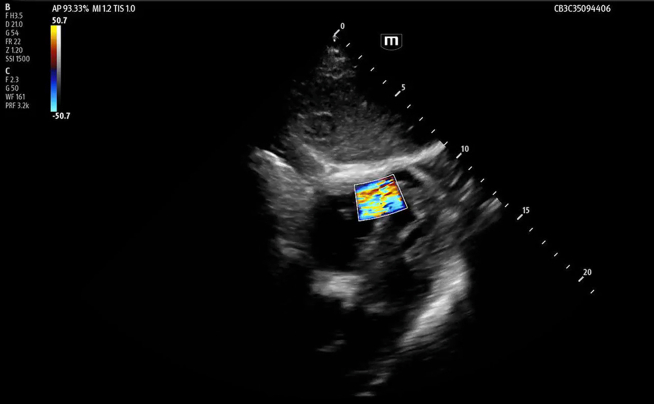
Point-of-care ultrasound revealing ventricular septal defect, apical view.

**Video 2:**
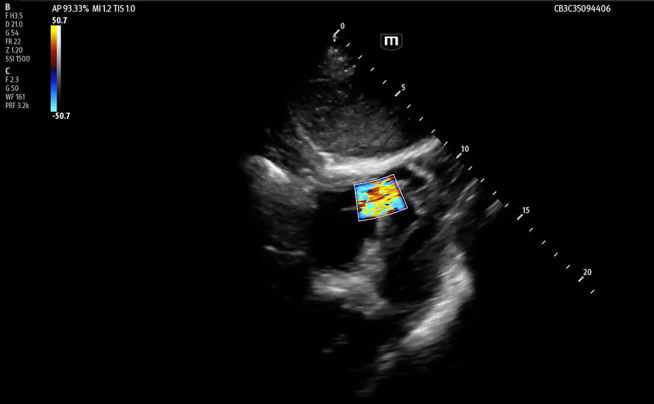
Point-of-care ultrasound demonstrating ventricular septal defect symptoms.

## Conflict of Interest

All authors have affirmed they have no conflicts of interest to declare.
